# Butyrate limits human natural killer cell effector function

**DOI:** 10.1038/s41598-023-29731-5

**Published:** 2023-02-15

**Authors:** Vanessa Zaiatz-Bittencourt, Fiona Jones, Miriam Tosetto, Caitriona Scaife, Gerard Cagney, Evan Jones, Glen A. Doherty, Elizabeth J. Ryan

**Affiliations:** 1 Centre for Colorectal Disease, St. Vincent’s University Hospital and School of Medicine, University College Dublin, Dublin 4, Ireland; 2 School of Biomolecular and Biomedical Science, Conway Institute of Biomedical and Biomolecular Sciences, University College Dublin, Dublin, Ireland; 3 Department of Biological Sciences, Faculty of Science and Engineering, University of Limerick, Limerick, Ireland; 4 Limerick Digital Cancer Research Centre, Health Research Institute, University of Limerick, Limerick, Ireland

**Keywords:** Extracellular signalling molecules, Innate lymphoid cells

## Abstract

The gut microbiota regulates chronic inflammation and has been implicated in the pathogenesis of a broad spectrum of disease including autoimmunity and cancer. Microbial short-chain fatty acids (SCFAs) e.g., butyrate have demonstrated immunomodulatory effects and are thought to be key mediators of the host-microbiome interaction. Here, we investigated the effect of butyrate on effector functions of blood derived human NK cells stimulated for 18 h with a combination of IL-12/IL-15, a potent mix of cytokines that drive NK cell activation. We show that butyrate has a strong anti-inflammatory effect on NK cells. NK cells cultured in the presence of butyrate expressed lower levels of activating receptors (TRAIL, NKp30, NKp44) and produced lower levels of cytokines (IFNγ, TNF-α, IL-22, granzyme B, granzyme A, perforin) in response to IL-12/IL-15. Butyrate restricted NK cell function by downregulation of mTORC1 activity, *c-Myc* mRNA expression and metabolism. Using a shotgun proteomic approach, we confirmed the effect of butyrate on NK cell cytokine signaling and metabolism and identified BRD2, MAT2A and EHD1 as downstream mediators of these effects. This insight into the immunomodulatory activity of butyrate on human NK cell function might help to develop new ways to limit NK cell function during chronic inflammation.

## Introduction

Gut microbial dysbiosis plays an important role in the pathogenesis of chronic inflammatory conditions such as inflammatory bowel disease (IBD), rheumatoid arthritis (RA), obesity and cancer^1^. Microbial metabolites such as short-chain fatty acids (SCFA) such as acetate, butyrate and propionate are produced through the fermentation of dietary fibers are known to have potent immunomodulatory activity^1^. SCFA can cross the intestinal epithelium and reach the lamina propria and peripheral blood where they can shape immune responses. Butyrate, a well-known SCFA is key to maintaining homeostasis in the gut^2^. Butyrate promotes both the anti-inflammatory effects of Treg cells and microbiocidal activity of macrophages^3–5^. Butyrate exerts these effects via different pathways depending on cell type. For example, butyrate can inhibit mammalian target of rapamycin complex 1 (mTORC1), act as a histone deacetylase (HDAC), modulate cell metabolism and stabilize HIF1-α, reducing the secretion of pro-inflammatory cytokines^5–9^. Despite many studies on the impact of SCFA in T cells, monocytes, and dendritic cells^4,5,7,8,10,11^, little is known about the impact of these microbial metabolites on human natural killer (NK) cell function. Here, our aim was to investigate the impact of butyrate treatment on human NK function focusing on cell metabolism, receptor expression and cytokine secretion.

NK cells act as a bridge between innate and adaptive immunity by secreting pro-inflammatory cytokines that activate and polarize other immune cells. In addition, NK cells can kill tumor cells or virally infected cells that have downregulated expression of MHC class I molecules on their surface^12^. In the peripheral blood, human NK cells can be subdivided into two subsets based on the surface expression of CD56, namely CD56^bright^ cells and CD56^dim^ cells. CD56^dim^ cells comprise 90% of the total NK cells in the human peripheral blood and are highly cytotoxic. CD56^bright^ cells are around 10% of the total NK cells in the peripheral blood and express high levels of cytokine receptors (IL-2R, IL-12R, IL-15R, IL-18R) and produce large quantities of cytokines e.g., IFN-γ^13^. NK cell cytotoxicity and cytokine production are tightly regulated by activatory/inhibitory NK cell receptors. Furthermore, optimal NK cell effector function relies on energy derived from glycolysis and oxidative metabolism^12^. We have previously demonstrated that peripheral blood NK cells obtained from patients with IBD have an unbalanced metabolic profile, with faulty mitochondria and reduced capacity to kill^14^.

In the present study, we report that butyrate constrains the activation of cytokine stimulated human NK cells. Butyrate reduced the expression of cell surface receptors and secretion of pro-inflammatory cytokines. Our results suggest this inhibitory effect is mediated by mTORC1, *c-Myc* and cell metabolism. Butyrate’s ability to restrain peripheral blood NK cells effector functions may explain its importance in maintaining immune homeostasis.

## Results

### Butyrate constrains the NK cell response to IL-12/IL-15, inhibiting both receptor expression and cytokine production

**Figure 1:**
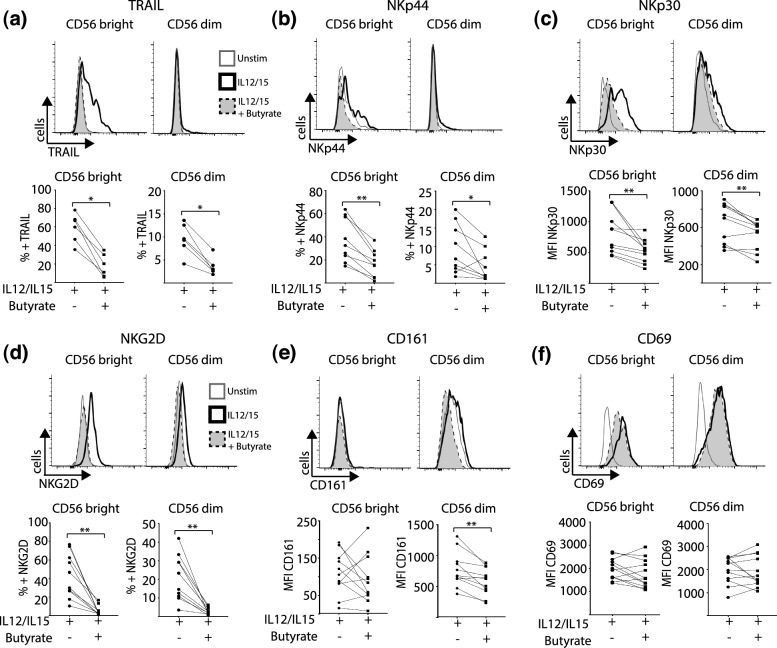
Butyrate regulates expression of NK cell receptors. PBMC were cultured for 18 h in media alone (unstim) or in the presence of IL-12 (30 ng/mL) and IL-15 (100 ng/mL). Butyrate (1 mM) was added where indicated for the duration of the experiment. Flow cytometry analysis was used to analyze NK cell receptor expression and results are displayed as either percentage of cell population or mean fluorescent intensity (MFI) Representative histograms (upper panel) and individual data points (lower panel) of CD56^bright^ cells and CD56^dim^ NK cells expressing (**a**) TRAIL (**b**) NKp44 (**c**) NKp30. (**d**) NKG2D (E) CD161 (F) CD69 (n = 6–12). Samples were compared using Wilcoxon signed rank test. *p < 0.05, **p < 0.01.

PBMCs were treated with IL-12/IL-15 for 18 h in the presence or absence of butyrate and the expression of a panel of NK cell receptors were analyzed by flow cytometry. We chose to evaluate expression of NK cell surface receptors related to cell activation, including TRAIL, NKp44, NKp30, NKG2D, CD161 and CD69. NK cells cultured with this cytokine combination for 18 h significantly upregulated their expression of these activatory NK cell receptors (Fig. 1a–f, Supplementary Fig. 1A–C). Addition of butyrate (1 mM) to the cultures suppressed upregulation of the following NK cell receptors by both CD56^bright^ and CD56^dim^ NK cells in response to IL-12/IL-15: TRAIL, NKp44, NKp30 and NKG2D (Fig. 1a–f, Supplementary Fig. 1A–C). Butyrate only modulated CD161 expressed by CD56^dim^ NK cells, as this is the subset which preferentially expresses this receptor (Fig. 1e)^15^. Interestingly, butyrate did not impact CD69 expression (Fig. 1f). We also tested the effect of acetate and propionate on NK cell activation using this model. Acetate (5–10 mM) did not significantly alter NK cell cytokine production or receptor expression in response to IL-12/15 stimulation (Supplementary Fig. 1F–I). While propionate (5–10 mM) behaved in a manner more like butyrate, inhibited IL-12/IL-15 induced IFN-γ, granzymeB, NKp30 and CD71 (Supplementary Fig. 1J–M).

**Figure 2:**
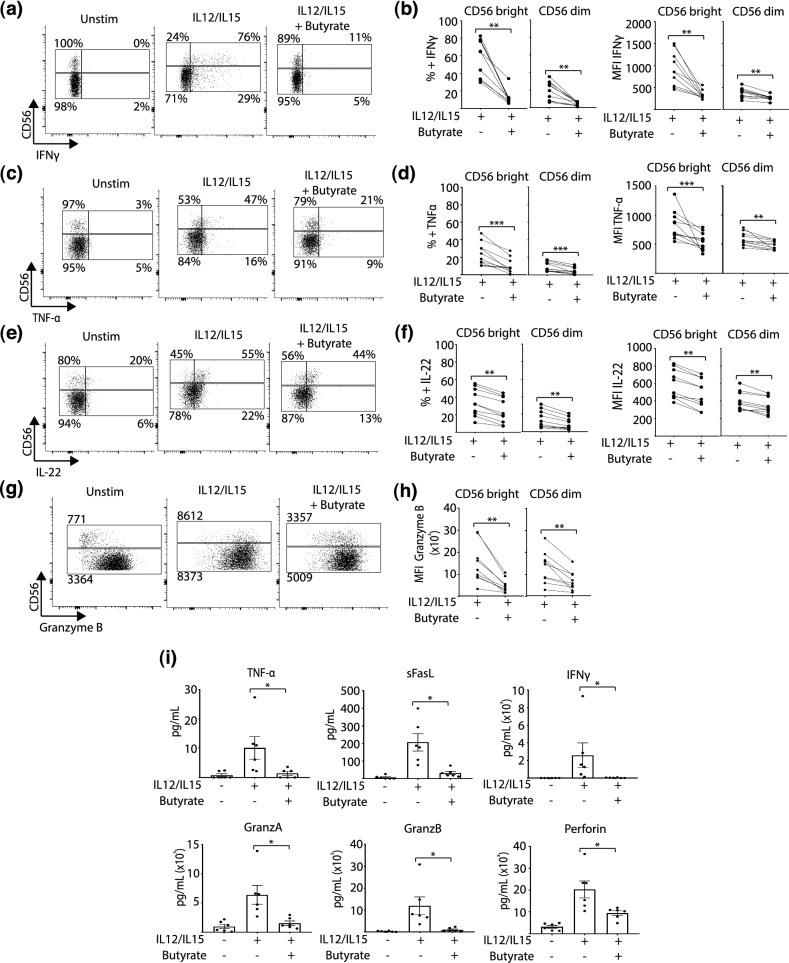
Butyrate impairs the production of inflammatory cytokines by human NK cells. PBMC were cultured for 18 h with or without IL-12 (30 ng/mL) and IL-15 (100 ng/mL). Butyrate (1 mM) was added where indicated for the duration of the experiment. Cytokine production was assessed by intracellular cytokine staining with Golgi-plug added to cultures for the final 3 h. Representative dot plots and individual paired data (with/without butyrate) of frequency and expression (MFI) of cytokines by CD56^bright^ cells (left panel) or CD56^dim^ cells (right panel) (n = 10) (**a**,**b**) IFNγ, (**c**,**d**) TNF-α, (**e**,**f**) IL-22, (**g**,**h**) Granzyme B. NK cells were purified by negative selection and cultured as described above. Cytokine concentration in the supernatant was measured using BioLegend’s LEGENDplex bead-based immunoassay. (**i**) TNF-α, sFasL (soluble Fas ligand), IFNγ, granzyme A, granzyme B, perforin (n = 6). Bars show the average value ± SEM. Samples were compared using the Wilcoxon signed rank test. *p < 0.05, **p < 0.01, ***p < 0.001.

Next, we tested whether butyrate could inhibit NK cell cytokine production (IFN-γ, TNF-α, IL-22, sFASL, granzyme B, granzyme A, perforin), in response to IL-12/IL-15 activation. The intracellular staining experiments clearly show NK cells cultured with IL-12/IL-15 produced IFNγ, TNF-α, IL-22 and granzyme B (Fig. 2a–h). Addition of butyrate to the cultures significantly inhibited the production of all the investigated cytokines (Fig. 2a–h). Similarly, our analysis of supernatant harvested from NK cells cultured with IL-12/IL-15 ± butyrate with the immune-bead multiplex assay clearly shows butyrate reduced secretion of TNF-α, soluble Fas ligand (sFasL), IFNγ, granzyme A, granzyme B and perforin (Fig. 2i). In terms of direct killing, NK cells treated with butyrate were able to kill standard K562 target cells efficiently as the non-treated NK cells (data not shown). Taken together, the results show that butyrate suppresses the response of human NK cells (both CD56^bright^ and CD56^dim^) to IL-12/IL-15, inhibiting both receptor expression and production of inflammatory cytokines.

### Butyrate regulates the expression of multiple proteins by cytokine stimulated NK cells

**Figure 3:**
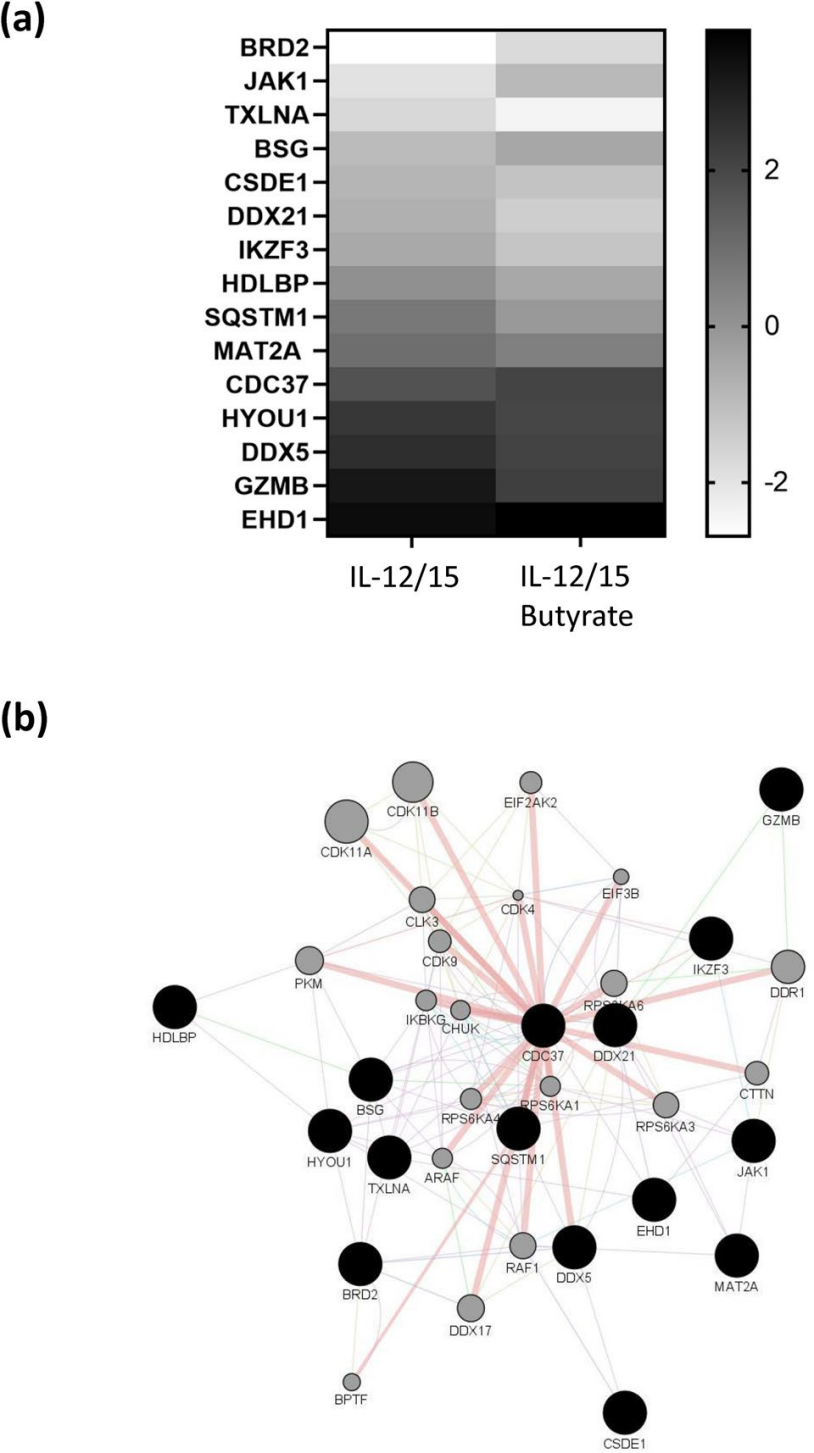
Effect of Butyrate on signaling pathways in NK cells. NK cells were purified by negative selection and cultured for 18 h with IL-12 (30 ng/mL) and IL-15 (100 ng/mL) with or without Butyrate (1 mM) (n = 6). (**a**) Differential protein expression was evaluated by mass spectrometry. 15 proteins were differentially expressed, p < 0.01. (**b**) The GeneMANIA Cytoscape plugin was used to predict protein interaction and construct a network. The list of 15 differentially expressed proteins were provided as a query (black nodes) and additional proteins were predicted to be related (grey nodes).

Shotgun proteomics analysis identified 15 differentially expressed proteins (p < 0.01) and 48 differentially expressed proteins (p < 0.05) following butyrate treatment of cytokine stimulated NK cells (Fig. 3a, Supplementary Table 1, “Differential protein expression” sheet). Five of the 15 identified proteins (p < 0.01) were upregulated following butyrate treatment (BRD2, JAK1, BSG, CDC37 and EHD1). Gene Ontology term analysis of the 15 highly differentially regulated proteins revealed an enrichment for terms related to NK cell function (regulation of IFN-γ-mediated signaling pathway) and cellular metabolism (ATP binding) (Supplementary Table 1, “Differential protein expression” and “GO Terms” sheet).

The relationships between the differentially expressed proteins were explored by constructing a protein–protein interaction (PPI) map using GeneMANIA (Fig. 3b). The functions in the PPI network include protein serine/threonine kinase activity, cyclin-dependent protein kinase activity, peptidyl-serine modification, regulation of translation, serine/threonine protein kinase complex, protein kinase complex, cellular response to interleukin-1, regulation of innate immune response, regulation of cytokine-mediated signaling pathway, modification-dependent protein binding (Supplementary Table 1, “Pathway Analysis” sheet).

### Butyrate inhibits key metabolic signaling pathways in NK cells

Next, we wished to determine if butyrate suppressed NK cell function by interfering with metabolic signaling pathways. NK cell activation in response to cytokine signals requires the mobilization of cellular energy. The mTORC1, c-Myc and HIF1-α dependent signaling pathways govern the metabolic requirements of NK cell activation^16–18^.

**Figure 4:**
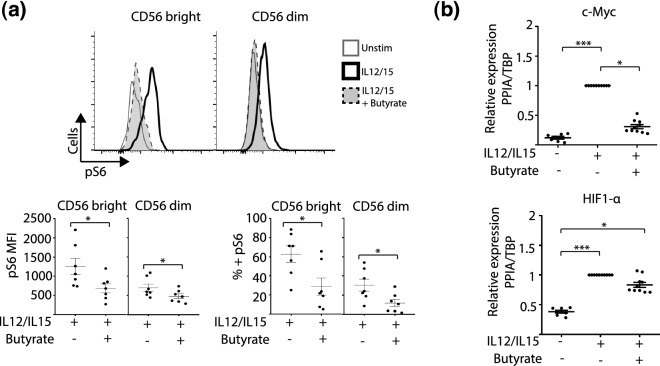
Butyrate regulates key metabolic signaling pathways in human NK cells. PBMC were cultured for 18 h in the absence or presence of IL-12 (30 ng/mL) with IL-15 (100 ng/mL). Butyrate (1 mM) was added for the duration of the experiment where indicated. (**a**) Phospho-S6 (pS6) (a surrogate marker for mTORC1 activation) was assessed by intracellular flow cytometry staining, gating on CD56^bright^ and CD56^dim^ NK cells. Representative histogram plots of pS6 levels (upper panel). Individual data points of pS6 expression (mean fluorescence intensity and percentage) by CD56^bright^ and CD56^dim^ NK cells are shown (lower panel) (n = 7), bar represents mean and error bars standard error of mean (SEM). (**b**) NK cells were purified by negative selection and cultured for 18 h in the absence or presence of IL-12 (30 ng/mL) and IL-15 (100 ng/mL). Butyrate (1 mM) was added where indicated for the duration of the experiment. Expression of *c-Myc* (upper panel) and *HIF-1α* (lower panel) was measured by real-time PCR and results were normalized relative to PPIA/TBP genes (n = 10). The bar represents the average value and error bars, SEM. Samples were compared using either a one-way ANOVA followed by a Kruskal–Wallis post hoc test or Wilcoxon’s signed rank test. *p < 0.05, **p < 0.01, ***p < 0.001.

First, we tested if butyrate could affect phosphorylation of S6, a direct mTORC1 substrate. IL-12/IL-15 treatment increased phospho-S6 levels in both CD56^bright^ cells and CD56^dim^ NK cell subsets. This agrees with previous work^13,16^. Interestingly, addition of butyrate to the cultures significantly reduced S6 phosphorylation in response to IL-12/IL-15 (Fig. 4a). This data indicates that butyrate can limit mTORC1 activation in response to IL-12/Il-15 in both CD56^bright^ and CD56^dim^ NK cell subsets.

We next investigated the ability of butyrate to impact expression of *c-Myc* and *HIF1-α*. Purified NK cells stimulated with IL-12/IL-15 expressed significantly higher levels of both *c-Myc* and *HIF1-α* compared to resting cells. Butyrate treatment significantly impaired *c-Myc* expression in response to cytokine treatment, bringing expression levels back to baseline. However, butyrate did not change expression of HIF1-α (Fig. 4b). This data indicates that butyrate inhibits mTORC1 and c-myc dependent signaling in NK cells.

### Butyrate represses the metabolic program of NK cells

**Figure 5:**
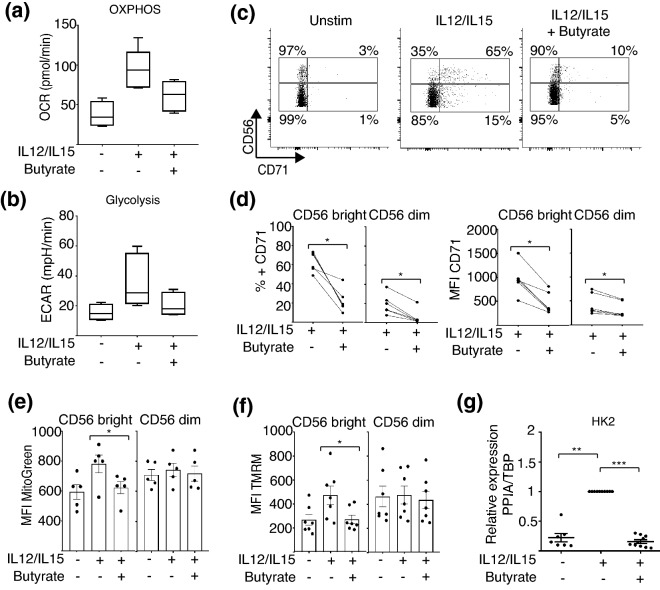
Butyrate impacts NK cell metabolic machinery. Purified NK cells were cultured for 18 h in the absence or presence of IL-12 (30 ng/mL) and IL-15 (100 ng/mL). Butyrate (1 mM) was added where indicated for the duration of the experiment. Measurement of cellular bioenergetics (respiratory activity) was performed using the Seahorse extracellular flux analyzer. (**a**) Oxygen consumption rate (OCR) and (**b**) extracellular acidification rate (ECAR), data displayed as boxplots with bar representing the mean and error bars the SEM (n = 5). PBMCs were cultured for 18 h in the absence or presence of IL-12 (30 ng/mL) and IL-15 (100 ng/mL). Butyrate (1 mM) was added where indicated for the duration of the experiment. CD71 expression by was determined by flow cytometry. (**c**) Representative dot plots and (**d**) individual paired data of CD71 expression by CD56^bright^ and CD56^dim^ NK cells, is shown (n = 6). (**e**) Mitochondrial mass of NK cells was measured by MitoTracker Green staining of PBMCs and gating on CD56^bright^ and CD56^dim^ cells. (**f**) Mitochondrial membrane potential analysis was performed by staining PBMC with TMRM (100 nM) for 30 min at 37 °C and gating on CD56^bright^ and CD56^dim^ NK cells. Individual datapoints are shown and bars represent mean and error bars, SEM (n = 5–7). (**g**) Purified NK cells were cultured with IL-12 (30 ng/mL) and IL-15 (100 ng/mL) ± Butyrate (1 mM) as described above. Expression of hexokinase 2 (*HK2*) was assessed by qRT-PCR (n = 7–10). Individual datapoints are shown, bars represent the average value ± SEM. Samples were compared using either a one-way ANOVA followed by a Kruskal–Wallis post hoc test or Wilcoxon’s signed rank test, as appropriate *p < 0.05, **p < 0.01, ***p < 0.001.

NK cells require mTORC1 activity to fuel their response to cytokine stimulation^13,16^. Our observation that butyrate inhibits mTORC1 activity suggests that its anti-inflammatory properties may be attributed to inhibition of NK cell metabolism. We undertook a series of experiments to investigate whether butyrate inhibited different aspects of NK cell metabolic function. Previous studies have shown that stimulated NK cells have increased rates of glycolysis and OXPHOS^16^. To investigate the impact of butyrate on these metabolic pathways, purified NK cells were stimulated with IL-12/IL-15 in the presence or absence of butyrate prior to metabolic analysis. Butyrate consistently diminished the rates of both OXPHOS (OCR, oxygen consumption rate) and glycolysis (ECAR, extracellular acidification rate) of the NK cells from 5 donors (Fig. 5a,b). However, the observed difference did not reach statistical significance (p = 0.06). We next investigated the expression of the nutrient receptor CD71 (transferrin receptor). It has been previously shown that increased CD71 expression is correlated with increased glycolysis and cell activity in immune cells, including NK cells^13,19^. Furthermore, CD71 is regulated by mTORC1 signaling pathway^16,20^. Resting NK cells have very low levels of CD71 expression which is increased in response to IL-12/IL-15 (Fig. 5c,d). Butyrate treatment consistently and significantly decreased the expression of CD71 in both CD56^bright^ cells and CD56^dim^ cells following activation by IL-12/IL-15 (Fig. 5c,d). As previously shown, CD71 is preferentially upregulated by CD56^bright^ NK cells in comparison to CD56^dim^ NK cells (Fig. 5c,d)^16^.

We next measured parameters of mitochondrial function in human NK cells, namely, mitochondrial membrane potential (MMP), mitochondrial mass and total cell reactive oxygen species (ROS) production by flow cytometry. Culture of NK cells for 18 h with IL-12/IL-15 increased mitochondrial mass and MMP of CD56^bright^ NK cells (Fig. 5e,f). This increase was significantly inhibited by butyrate treatment (Fig. 5e,f). These changes were not observed in CD56^dim^ NK cells and we did not observe any difference in ROS production (Supplementary Fig. 2A).

Finally, we assessed impact of butyrate on the gene expression of three important metabolic enzymes: Hexokinase 2 (*HK2*), ATP synthase F1 subunit beta (*ATP5B*) and lactate dehydrogenase (*LDHA)*. Expression of *HK2*, the first enzyme in glycolysis, was significantly increased following IL-12/IL-15 treatment compared to resting NK cells (Fig. 5g). Butyrate treatment significantly inhibited the upregulation of *HK2* expression in response to cytokine stimulation (Fig. 5g). Neither cytokines nor butyrate treatment altered lactate dehydrogenase (*LDHA)* expression, the enzyme that catalyzes the conversion of lactate to pyruvate (Supplementary Fig. 2b), or the expression of ATP synthase F1 subunit beta (*ATP5B*), which is a subunit of mitochondrial ATP synthase responsible for ATP synthesis (Supplementary Fig. 2c). Taken together, these findings indicate that the inhibitory effect of butyrate correlates with a decline in mitochondrial function, glycolysis and OXPHOS mainly through inhibition of *HK2*, which in turn likely impairs human NK cell cellular fitness and activation.

## Discussion

The importance of NK cells’ role in the regulation of innate and adaptive immunity is undoubted. NK cells are not only important in the fight against cancer and viral infection, but also orchestrate inflammatory responses^21^. The role of the microbiome in regulation of human health and immunity is equally clear^3^. Yet, we do not fully understand how variation in the microbiome/microbial metabolites impacts NK cell biology.

We tested acetate, propionate, and butyrate, as they are all important SCFA produced by gut-resident microbiota. At the concentrations tested, both butyrate and propionate acted in a similar manner and could blunt the response of NK cells to IL-12/IL-15 stimulation. However, under these conditions, acetate did not significantly impact on NK cell receptor expression or cytokine secretion. These differential responses could be mediated by different receptor usage. Butyrate and propionate are known to signal through the G-protein coupled receptor, the free fatty acid receptor 3 (FFA3) while acetate preferentially signals though free fatty acid receptor 2 (FFAR2)^22^. Similar results were observed in macrophages, where propionate and butyrate but not acetate strongly inhibited inflammatory response^23^. Interestingly, butyrate and propionate also possess anti-inflammatory properties in other cell types such as iPSC-derived intestinal epithelia and periodontal tissue^24,25^. Butyrate was found to be the SCFA with the most consistent effects on NK cell metabolism and activation receptors at the concentrations used (1 mM, 2.5 mM and 5 mM). In the present study we show that butyrate, a SCFA derived from the fermentation of fiber by the gut microbiota, inhibits many aspects of human NK cell effector functions.

The combination of IL-12/IL-15 activates NK cells, promoting the metabolic changes required to fuel increases in receptor expression and cytokine secretion^16^. Butyrate significantly dampens NK cells’ response to this cytokine stimulus. While we found butyrate had no adverse effect on cell viability (at concentration used). NK cells treated with butyrate did not upregulate expression of TRAIL, NKp44, NKp30, NKG2D on their cell surface in response to cytokine stimulation. Butyrate only reduced CD161 expression by CD56^dim^ cells, as this subset express the highest levels of this receptor^15^. However, butyrate treatment had no effect on CD69 expression. Besides receptor expression, butyrate also significantly inhibited the production of the pro-inflammatory cytokines TNF-α, IFN-γ, IL-22, soluble Fas ligand, granzyme A, granzyme B and perforin. These cytokines are key immune mediators and dysregulation of these proteins can be linked to cancer progression or autoimmune disease pathogenesis^26–28^. There is still controversy surrounding the effects of butyrate on diseases like obesity^29^ however, it is quite clear that NK cells from obese patients are defective^30^. Associating butyrate concentration with NK cell phenotype and function would provide insight into why obese patients have defective NK cells and a higher cancer risk. Butyrate treatment had no effect on the ability of NK cells to kill the leukemic K562 cell line^31^. Moreover, CD56^dim^ NK cells still express high quantities of Granzyme B after butyrate treatment. For further understanding of butyrate's impact on NK cell killing, other cell lines should be tested in addition to K562. The impact of butyrate on NK cell exhaustion could also be tested by performing long-term killing assays.

Butyrate may impact NK cell gene expression by interfering with histone acetylation, protein folding or blocking the expression of NF-kB^32^ or IFN-γ-induced JAK1^33^. Using shotgun proteomics analysis of cytokine stimulated NK cells in absence or presence of butyrate allowed us to identify several other potential mechanisms that may underpin the suppression of cytokine secretion observed following butyrate treatment. We found that NK cells treated with butyrate expressed higher levels of the BET bromodomain containing protein, BRD2. BRD2 controls inflammatory cytokine production by NK cells^34^. We also found that NK cells differentially express the ubiquitin-binding selective autophagy receptor p62/SQSTM1, and the RNA helicase DDX21 following butyrate treatment. Both molecules have been implicated in regulation of IFN^35,36^.

NK cells must mobilize energy to support their effector functions. mTORC1 is a key regulator of NK cell metabolism^13^. Following cytokine stimulation NK cells quickly increase mTORC1 activity, which is maintained for long periods of time, suggesting that mTORC1 is important for sustained NK cell effector functions^16^. Butyrate significantly inhibited mTORC1 activity consequently impacting NK cell metabolism and function. Similarly, butyrate also modulates mTORC1 activity and metabolism of other cell types including CD8^+^T cells, macrophages, and non-immune cells^5,8,37,38^.

We also demonstrated that butyrate-mediated inhibition of NK cells was through direct downregulation of *c-Myc* expression. c*-Myc* is important for cell development, proliferation, tumor surveillance and cellular metabolism^39,40^. c-Myc is downstream of the IL-15 signaling pathway, which regulates NK cell survival, cytotoxicity, and cytokine secretion^41,42^. Peripheral blood NK cells obtained from patients with cancer have limited expression of c-Myc which correlates with dysregulated function^43^. The mTORC1-c-MYC axis controls expression of the metabolic enzyme methionine adenosyltransferase 2α (MAT2A). MAT2A activity is required to support protein synthesis and tumour growth^44^. Interestingly, we found that NK cells have lower expression of this enzyme following butyrate treatment. The attenuation of mTORC1 signaling and *c-Myc* observed in NK cells treated with butyrate suggests that the anti-inflammatory effects may be indirectly attributed to inhibition of NK cell metabolism.

We detected that butyrate negatively impacted both glycolysis and OXPHOS. Importantly, butyrate significantly inhibited the expression of *HK2,* the first enzyme in the glycolytic pathway, which fits with the observed reduction in NK cell metabolism^45^. CD71 is a marker of NK cell activation and an indirect marker of activated metabolic machinery^16^. We show that following treatment with butyrate both CD56^bright^ and CD56^dim^ NK cell subsets expressed lower levels of the transferrin receptor, CD71. However, mitochondrial mass and polarization were mainly affected in the CD56^bright^ NK cell subset, which supports the rationale that CD56^bright^ NK cells are more dependent of mitochondrial function then their CD56^dim^ counterparts^13,14^. Our proteomic analysis showed that butyrate treatment of NK cells modulated expression of EH Domain-Containing Protein 1 (EHD1). EHD1 is an ATPase and endocytic protein that regulates mitochondrial fission^46^. Together these data suggest butyrate plays an important role in regulating mitochondrial dynamics in NK cells.

While glucose is the most efficient fuel source for cell energy production, cells can also use amino acids and fatty acids^45^. At the concentration of butyrate used in this study, NK cells may still be able to generate sufficient energy from these alternative sources to allow them to maintain viability. We found that butyrate treatment of NK cells modulated their expression of the Hsp90 co-chaperone Cdc37 which has been implicated in orchestrating the cellular response to glucose starvation in yeast by regulating transcription factor and kinase activity^47^. Understanding the strategies NK cells use to survive under metabolic stress may enable the development of more robust cell therapies^48^.

In summary, our findings indicate that butyrate is a strong inhibitor of human NK cell activation. Our work clearly shows that butyrate treatment modulates NK cell receptor expression, cytokine production and metabolism in response to cytokine activation. These findings may explain in part the anti-inflammatory properties of butyrate producing bacteria.

## Materials and methods

### Cell purification and cell culture

Blood samples were obtained from healthy adult volunteers (using BD Vacutainer tubes with Lithium Heparin as anti-coagulant) or heparinized blood donations were obtained from haemochromatosis patients following routine venesection. Informed consent was obtained from all participants and the protocol was approved by the St. Vincent’s University Hospital research and ethics committee. All experiments were performed in accordance with relevant guidelines and regulations. Peripheral blood mononuclear cells (PBMC) were isolated by density gradient centrifugation over Lymphoprep (StemCell Technologies), NK cells were purified by negative selection using magnetic cell sorting using the EasySep Human NK Cell Isolation kit following the manufacturer’s instructions (Stemcell Technologies). PBMC (5 × 10^6^ cells/mL) or purified NK cells (2 × 10^6^ cells/mL) were cultured in RPMI 1640 Glutamax medium supplemented with 10% fetal bovine serum (Gibco), 1% Penicillin/Streptomycin (Invitrogen) and incubated at 37 °C in a humidified atmosphere of 5% CO_2_ in air. PBMC or purified NK cells were stimulated for 18 h with recombinant IL-12 (Miltenyi Biotech) (30 ng/mL) in combination with IL-15 (Miltenyi Biotech) (100 ng/mL) in the presence or absence of butyrate (1–5 mM) (Sigma Aldrich, cat 303410).

We tested three different concentrations of butyrate in cultures of NK cells: 1 mM, 2.5 mM and 5 mM (Supplementary Fig. 1D,E). Additionally, we tested 5 mM, 7 mM, and 10 mM concentrations of acetate (Supplementary Fig. 1F–I) and propionate (Supplementary Fig. 1J–M) as lower concentrations (1 mM, 2.5 mM and 5 mM) did not show any impact on NK function (data not shown).

We selected the physiological butyrate concentration (1 mM) to execute our experiments as it is considered the most important SCFA. Butyrate at 1 mM had no impact on NK cell viability (Supplementary Fig. 1O).

### Flow cytometry

The following fluorochrome-conjugated monoclonal antibodies were used in this study: CD56 (REA196, NCAM 16.2), CD3 (SK7), CD71(M-A172); TRAIL (REA1113); NKG2D (1D11), NKp30 (P30-15), NKp44 (REA1163), CD69 (FN50), CD161 (HP-3G10), Granzyme B (QA18A28), IFN-γ (4S.B3), IL-22 (2G12A41), TNF-α (MAb11) (Biolegend, Miltenyi Biotech or BD Bioscience). For surface staining, cells were washed and stained for 20 min at 4 °C with saturating concentrations of antibodies. For the intracellular detection of cytokines, Golgi Plug (BD Bioscience) was added to cells for 3 h, cells were fixed and permeabilized using cytofix/cytoperm (BD Bioscience) according to the manufacturer’s instructions. Cells were stained for 30 min at 4 °C with saturating concentrations of antibodies. Live dead staining (Live/Dead Aqua, ThermoFisher) was used to exclude dead cells and to calculate cell viability.

Gating strategy: initial selection of lymphocyte population was performed using a forward/sideward scatterplot (FSC/SSC). The first gate was set around lymphocytes population. Doublets were excluded by gating forward scatter width (FSC-W) and forward scatter area (FSC-A). Live cells were gated according to FSC-A and SSC-A; and NK cells were identified as CD56^+^ CD3^-^ cells. (Supplementary Fig. 1N).

Samples were acquired by FACS Canto II (Becton Dickinson) and analyzed by FlowJo software (Flowjo/BD).

### Cytotoxicity assay

PBMC from 3 different donors were seeded at different Effector to Target (E:T) ratios (20:1, 10:1, 5:1, 1:1) in duplicate and stimulated for 18 h with recombinant IL-12 (Miltenyi Biotech) (30 ng/mL) in combination with IL-15 (Miltenyi Biotech) (100 ng/mL) in the presence or absence of butyrate (1 mM, Sigma Aldrich, cat 303410). On the next day, K562 cells were counted and washed thoroughly out of media using phosphate buffered saline (PBS). The cells were resuspended at a concentration of 1 × 10^6^ cells/mL in PBS + 2.5 µM Tag-IT Violet (Biolegend, cat 425101). Following 10 min incubation in a 37 °C, 5% CO_2_ incubator, the labelled K562 cells were thoroughly washed in complete culture media (RPMI 1640 Glutamax medium supplemented with 10% fetal bovine serum, Gibco; 1% Penicillin/Streptomycin, Invitrogen). K562 cells were resuspended at a concentration of 0.3 × 10^6^ cells/mL and co-cultured with PBMC for the last 4 h of incubation. After the incubation, cells were washed and stained with Live Dead Near-IR (Biolegend) and CD56-PE antibody (Miltenyi Biotech) for 10 min. After surface staining, cells were washed and resuspended in PBS + 2% serum.

Samples were acquired by FACS Canto II (Becton Dickinson) and the percentage killing of K562 was determined by FlowJo software (Flowjo/BD).

### Mitochondria mass, mitochondria membrane potential and cellular reactive oxygen species (ROS)

Mitochondrial mass was measured via staining of cells for 30 min with MitoTracker Green (100 nM, ThermoFisher), mitochondria membrane potential analysis was performed by staining cells with TMRM (tetramethylrhodamine, methyl ester, 100 nM, ThermoFisher) for 30 min and cellular reactive oxygen species (ROS) was measured via staining cells for 30 min with CellROX Green (500 nM, ThermoFisher). All samples were incubated at 37 °C in a humidified atmosphere of 5% CO_2_ in air. After washing, cells were stained with surface markers (CD56 and CD3) and live-dead aqua dye (BD Biosciences) was used to exclude dead cells. Samples were acquired by FACS Canto II (BD Biosicence) and analyzed by FlowJo software (Flowjo/BD).

### Metabolism analysis

Measurement of cellular bioenergetics (respiratory activity) was performed using the Seahorse extracellular flux analyzer (Agilent). In brief, 3 × 10^5^ purified NK cells were allowed to adhere to an 8-well XF cell culture microplate (Seahorse Biosciences) coated with Cell-Tak (Corning). Sequential measurements of oxygen consumption rate (OCR, representative of OXPHOS) and extracellular acidification rate (ECAR, representative of glycolysis) were made after the addition of the inhibitors oligomycin (2 µM, ATP synthase blocker, Sigma-Aldrich, cat O4876), carbonyl cyanide-4-(trifluoromethoxy) phenylhydrazone (FCCP, 0.5 µM, Sigma-Aldrich, cat C2920), rotenone (100 nM, inhibitor of mitochondrial complex I, Sigma-Aldrich, cat R8875) plus antimycin A (4 µM, inhibitor of mitochondrial complex III, Sigma-Aldrich, cat A8674) and 2-deoxyglucose (2DG, 30 mM, glycolysis inhibitor, Fischer Scientific, cat 10152950).

### Cytokine measurement

Purified NK cells (2 × 10^6^ cells/mL) were cultured for 18 h with recombinant IL-12 (30 ng/mL) in combination with IL-15 (100 ng/mL) in the presence or absence of butyrate (1 mM). Supernatants were collected and assessed for cytokine content with a LEGENDplex human NK/CD8 cell panel (BioLegend, cat 740267) according to the manufacturer’s guidelines. Allowing quantification of TNFα, soluble Fas ligand (sFasL), IFNγ, granzyme A, granzyme B and perforin secreted by the NK cells. Samples were acquired using a FACS Canto II and data analyzed using LEGENDplex Data analysis software (BioLegend).

### Gene expression analysis

Purified NK cells were cultured for 18 h in the absence or presence of IL-12 (30 ng/mL) with IL-15 (100 ng/mL), with or without Butyrate (1 mM). RNA was isolated using the E.Z.N.A. Total RNA kit I (Omega Bio-tek, USA) and reverse transcribed using the qScript cDNA SuperMix Kit (Quantabio) following the recommended protocols. Each reverse transcription reaction was performed in a final volume of 20 µl using 200 ng of RNA for each sample. The cDNA samples obtained were stored at − 20 °C.

Gene expression was assessed using Applied Biosystems TaqMan real-time PCR assays. TaqMan universal PCR MasterMix (cat 4305719, Applied Biosystems) was used as per protocol provided. Samples were run on a LightCycler LC480 PCR machine (Roche Molecular Systems, Inc).

The relative mRNA expression (Cp value) of each sample was calculated with LightCycler software using the 2-∆∆Cp method. The threshold cycle was normalized to the reference genes (*PPIA* and *TBP*), using Microsoft Excel and GraphPad Prism software (version 8.0; GraphPad Software, Inc, La Jolla, CA). The IL-12/IL-15 stimulated sample was designated as the calibrator for each donor. Using the 2 -∆∆Cp method the data are presented as the fold change in gene expression normalised to an endogenous reference gene and relative to the IL-12/IL-15 stimulated cells (for this sample ∆∆Cp equals zero and 2^0^ = 1).

### Proteomics

Purified NK cells (2 × 10^6^ cells) were cultured for 18 h in the presence or absence of IL-12 (30 ng/mL) with IL-15 (100 ng/mL) with or without Butyrate (1 mM). Cells were pelleted, re-suspended in 40 µL urea lysis buffer (6 M-Urea; 0.1 M-Tris–Cl pH 8.0) containing protease inhibitors (complete, Mini Protease Inhibitor Cocktail, Roche, 11836153001), sonicated briefly on ice using a hand-held microprobe and incubated for 30 min. Protein content was estimated using a modified Bradford assay^49^. For mass spectrometry a volume equivalent to 20 µg of each sample was reduced in the presence of Dithiothreitol (final concentration 10 mM, 60 min at 30 °C) and alkylated in the dark in the presence of Iodoacetamide (final concentration of 30 mM for 30 min at room temperature) before diluting with five volumes of 50 mM Triethyl ammonium bicarbonate, to reduce the urea content to < 2 M. Trypsin (Promega, V5111) was added at a ratio of 1:50 enzyme:protein and samples were incubated at 37 °C overnight. Digestion was stopped the following morning with the addition of TFA (trifluoroacetic acid) to each sample. Tryptic digests were de-salted using C18 ZipTips (Millipore, Ref ZTC18S960) prior to resuspension in 0.1% formic acid to a concentration of 40 ng/µL.

Samples were run on a timsTOF Pro mass spectrometer (Bruker Daltonics, Bremen, Germany) coupled to a nanoElute (Bruker Daltonics, Bremen, Germany) ultra-high pressure nanoflow chromatography system (UHPnLC). The timsTOF Pro mass spectrometer was operated in positive ion polarity with TIMS (Trapped Ion Mobility Spectrometry) and PASEF (Parallel Accumulation Serial Fragmentation) modes enabled. The accumulation and ramp times for the TIMS were both set to 100 ms., with an ion mobility (1/k0) range from 0.62 to 1.46 Vs/cm. Spectra were recorded in the mass range from 100 to 1700 m/z. The precursor (MS) Intensity Threshold was set to 2500 and the precursor Target Intensity set to 20,000. Each PASEF cycle consisted of one MS ramp for precursor detection followed by 10 PASEF MS/MS ramps, with a total cycle time of 1.16 s. The peptides were separated on a reversed-phase C18 Aurora column (25 cm × 75 μm ID, C18, 1.6 μm; IonOpticks, Australia) at a constant flow rate of 250 nL/min and an increasing acetonitrile gradient. Mobile phases were 0.1% (v/v) formic acid in water (phase A) and 0.1% (v/v) formic acid in acetonitrile (phase B). The peptides were separated by a gradient starting from 2% of mobile phase B and increased linearly to 30% for 57 min. This was then stepped up to 95% of mobile phase B where it was maintained for 7 min, and finally decreased to 2% where it was held for 2 min to wash and re-equilibrate the column. An injection volume was 5 μL throughout the run, equivalent to an estimated 200 ng per sample.

Protein identification and label free quantitation (LFQ) was performed on the Bruker data files using MaxQuant (v 1.6.14.0) and searched against the Uniprot Human Reference Proteome database (downloaded 2019). A contaminants and reverse database were also included in the search. For searching, the default variable modifications of oxidation of methionine, and acetylation of protein N-termini, and the fixed modification carbamidomethylation of cysteine were used. Two missed cleavages were allowed for and both protein and peptide FDRs were set at 0.01. The ‘match between runs’ option was also selected.

LFQ Intensity values were used for the statistical analysis in Perseus (v 1.6.14). Data were first filtered to remove reverse hits and contaminants. Proteins that did not appear in a minimum of 70% of the samples were excluded from the quantitation. LFQ Intensity values were log2 transformed, missing values imputed from a normal distribution, and column normalization was performed by subtracting the median value. A Student’s t-test was performed on the grouped samples and proteins were deemed to be significant at a value of p < 0.01. This yielded a list of 15 proteins. This list was submitted to Genemania (Cytoscape version 3.8.2) to construct a composite gene–gene functional interaction network. The resulting network also imputes the genes most related to the original list. Gene Ontology pathway enrichment analysis was performed using the DAVID database (https://david.ncifcrf.gov, last accessed April 2022)^50^.

### Statistical analysis

GraphPad Prism (version 8.0; GraphPad Software) was used for statistical analysis. Data were compared using either a one-way ANOVA followed by a Kruskal–Wallis post hoc test or non-parametric paired data was analyzed using Wilcoxon Rank test, as appropriate.

## Supplementary Information

Supplementary Tables.

Supplementary Figure 1.

Supplementary Figure 2.

## Data Availability

The data underlying this article are available in the article and in its online supplementary material.

## References

[1] Armstrong H, Bording-Jorgensen M, Dijk S, Wine E (2018). The complex interplay between chronic inflammation, the microbiome, and cancer: Understanding disease progression and what we can do to prevent it. Cancers.

[2] Parada Venegas D (2019). Short chain fatty acids (SCFAs)-mediated gut epithelial and immune regulation and its relevance for inflammatory bowel diseases. Front. Immunol..

[3] Postler TS, Ghosh S (2017). Understanding the holobiont: How microbial metabolites affect human health and shape the immune system. Cell Metab..

[4] Arpaia N (2013). Metabolites produced by commensal bacteria promote peripheral regulatory T-cell generation. Nature.

[5] Schulthess J (2019). The short chain fatty acid butyrate imprints an antimicrobial program in macrophages. Immunity.

[6] Trompette A (2018). Dietary fiber confers protection against flu by shaping Ly6c(-) patrolling monocyte hematopoiesis and CD8(+) T cell metabolism. Immunity.

[7] Bachem A (2019). Microbiota-derived short-chain fatty acids promote the memory potential of antigen-activated CD8(+) T cells. Immunity.

[8] Luu M (2018). Regulation of the effector function of CD8(+) T cells by gut microbiota-derived metabolite butyrate. Sci. Rep..

[9] Yang W (2020). Intestinal microbiota-derived short-chain fatty acids regulation of immune cell IL-22 production and gut immunity. Nat. Commun..

[10] Kaisar MMM, Pelgrom LR, van der Ham AJ, Yazdanbakhsh M, Everts B (2017). Butyrate conditions human dendritic cells to prime type 1 regulatory T cells via both histone deacetylase inhibition and G protein-coupled receptor 109A signaling. Front. Immunol..

[11] Nastasi C (2015). The effect of short-chain fatty acids on human monocyte-derived dendritic cells. Sci. Rep..

[12] Vivier E (2011). Innate or adaptive immunity? The example of natural killer cells. Science.

[13] Keating SE (2016). Metabolic reprogramming supports IFN-gamma production by CD56bright NK cells. J. Immunol..

[14] ZaiatzBittencourt V, Jones F, Tosetto M, Doherty GA, Ryan EJ (2021). Dysregulation of metabolic pathways in circulating natural killer cells isolated from inflammatory bowel disease patients. J. Crohns Colitis.

[15] Kurioka A (2018). CD161 defines a functionally distinct subset of pro-inflammatory natural killer cells. Front. Immunol..

[16] Zaiatz-Bittencourt V, Finlay DK, Gardiner CM (2018). Canonical TGF-beta signaling pathway represses human NK cell metabolism. J. Immunol..

[17] Swaminathan S (2020). MYC functions as a switch for natural killer cell-mediated immune surveillance of lymphoid malignancies. Nat. Commun..

[18] Coulibaly A (2021). STAT3 governs the HIF-1alpha response in IL-15 primed human NK cells. Sci. Rep..

[19] MacIver NJ, Michalek RD, Rathmell JC (2013). Metabolic regulation of T lymphocytes. Annu. Rev. Immunol..

[20] Siska PJ (2017). Mitochondrial dysregulation and glycolytic insufficiency functionally impair CD8 T cells infiltrating human renal cell carcinoma. JCI Insight.

[21] Gianchecchi E, Delfino DV, Fierabracci A (2018). NK cells in autoimmune diseases: Linking innate and adaptive immune responses. Autoimmun. Rev..

[22] Kimura I, Ichimura A, Ohue-Kitano R, Igarashi M (2020). Free fatty acid receptors in health and disease. Physiol. Rev..

[23] Wu YL (2022). Propionate and butyrate attenuate macrophage pyroptosis and osteoclastogenesis induced by CoCrMo alloy particles. Mil. Med. Res..

[24] Grouls M (2022). Differential gene expression in iPSC-derived human intestinal epithelial cell layers following exposure to two concentrations of butyrate, propionate and acetate. Sci. Rep..

[25] Santos AFP, Cervantes LCC, Panahipour L, Souza FA, Gruber R (2022). Proof-of-principle study suggesting potential anti-inflammatory activity of butyrate and propionate in periodontal cells. Int. J. Mol. Sci..

[26] Boivin WA, Cooper DM, Hiebert PR, Granville DJ (2009). Intracellular versus extracellular granzyme B in immunity and disease: Challenging the dogma. Lab Investig..

[27] Rossin A, Miloro G, Hueber AO (2019). TRAIL and FasL functions in cancer and autoimmune diseases: Towards an increasing complexity. Cancers.

[28] Mortier E, Ma A, Malynn BA, Neurath MF (2020). Modulating cytokines as treatment for autoimmune diseases and cancer. Front. Immunol..

[29] Liu H (2018). Butyrate: A double-edged sword for health?. Adv. Nutr..

[30] O'Shea D, Hogan AE (2019). Dysregulation of natural killer cells in obesity. Cancers.

[31] Anfossi N (2006). Human NK cell education by inhibitory receptors for MHC class I. Immunity.

[32] Gallinari P, Di Marco S, Jones P, Pallaoro M, Steinkuhler C (2007). HDACs, histone deacetylation and gene transcription: From molecular biology to cancer therapeutics. Cell Res..

[33] Klampfer L, Huang J, Swaby LA, Augenlicht L (2004). Requirement of histone deacetylase activity for signaling by STAT1. J. Biol. Chem..

[34] Cribbs AP (2021). Dissecting the role of BET bromodomain proteins BRD2 and BRD4 in human NK cell function. Front. Immunol..

[35] Prabakaran T (2018). Attenuation of cGAS-STING signaling is mediated by a p62/SQSTM1-dependent autophagy pathway activated by TBK1. EMBO J..

[36] Wu W (2021). Caspase-dependent cleavage of DDX21 suppresses host innate immunity. MBio.

[37] Qiao CM (2020). Sodium butyrate causes alpha-synuclein degradation by an Atg5-dependent and PI3K/Akt/mTOR-related autophagy pathway. Exp. Cell Res..

[38] Cao M, Zhang Z, Han S, Lu X (2019). Butyrate inhibits the proliferation and induces the apoptosis of colorectal cancer HCT116 cells via the deactivation of mTOR/S6K1 signaling mediated partly by SIRT1 downregulation. Mol. Med. Rep..

[39] Cichocki F (2009). The transcription factor c-Myc enhances KIR gene transcription through direct binding to an upstream distal promoter element. Blood.

[40] Marchingo JM, Sinclair LV, Howden AJ, Cantrell DA (2020). Quantitative analysis of how Myc controls T cell proteomes and metabolic pathways during T cell activation. Elife.

[41] Bianchi T, Gasser S, Trumpp A, MacDonald HR (2006). c-Myc acts downstream of IL-15 in the regulation of memory CD8 T-cell homeostasis. Blood.

[42] Carson WE (1994). Interleukin (IL) 15 is a novel cytokine that activates human natural killer cells via components of the IL-2 receptor. J. Exp. Med..

[43] Zakiryanova GK (2019). Abnormal expression of c-Myc oncogene in NK cells in patients with cancer. Int. J. Mol. Sci..

[44] Villa E (2021). mTORC1 stimulates cell growth through SAM synthesis and m(6)A mRNA-dependent control of protein synthesis. Mol. Cell.

[45] O'Brien KL, Finlay DK (2019). Immunometabolism and natural killer cell responses. Nat. Rev. Immunol..

[46] Farmer T (2017). Control of mitochondrial homeostasis by endocytic regulatory proteins. J. Cell Sci..

[47] Rodkaer SV (2014). Quantitative proteomics identifies unanticipated regulators of nitrogen- and glucose starvation. Mol. Biosyst..

[48] Choi C, Finlay DK (2021). Optimising NK cell metabolism to increase the efficacy of cancer immunotherapy. Stem Cell Res. Ther..

[49] Ramagli L, Rodriguez L (1985). Quantitation of microgram amounts of protein in two-dimensional polyacrtlamide gel electrophoresis sample buffer. Electrophoresis.

[50] Huang DW (2007). DAVID Bioinformatics Resources: Expanded annotation database and novel algorithms to better extract biology from large gene lists. Nucleic Acids Res..

