# Supplementary figures and images for: Butyrate limits human natural killer cell effector function
Source: Sci Rep. 2023 Feb 15;13:2715. doi: 10.1038/s41598-023-29731-5 (PMC9932090; doi:10.1038/s41598-023-29731-5)

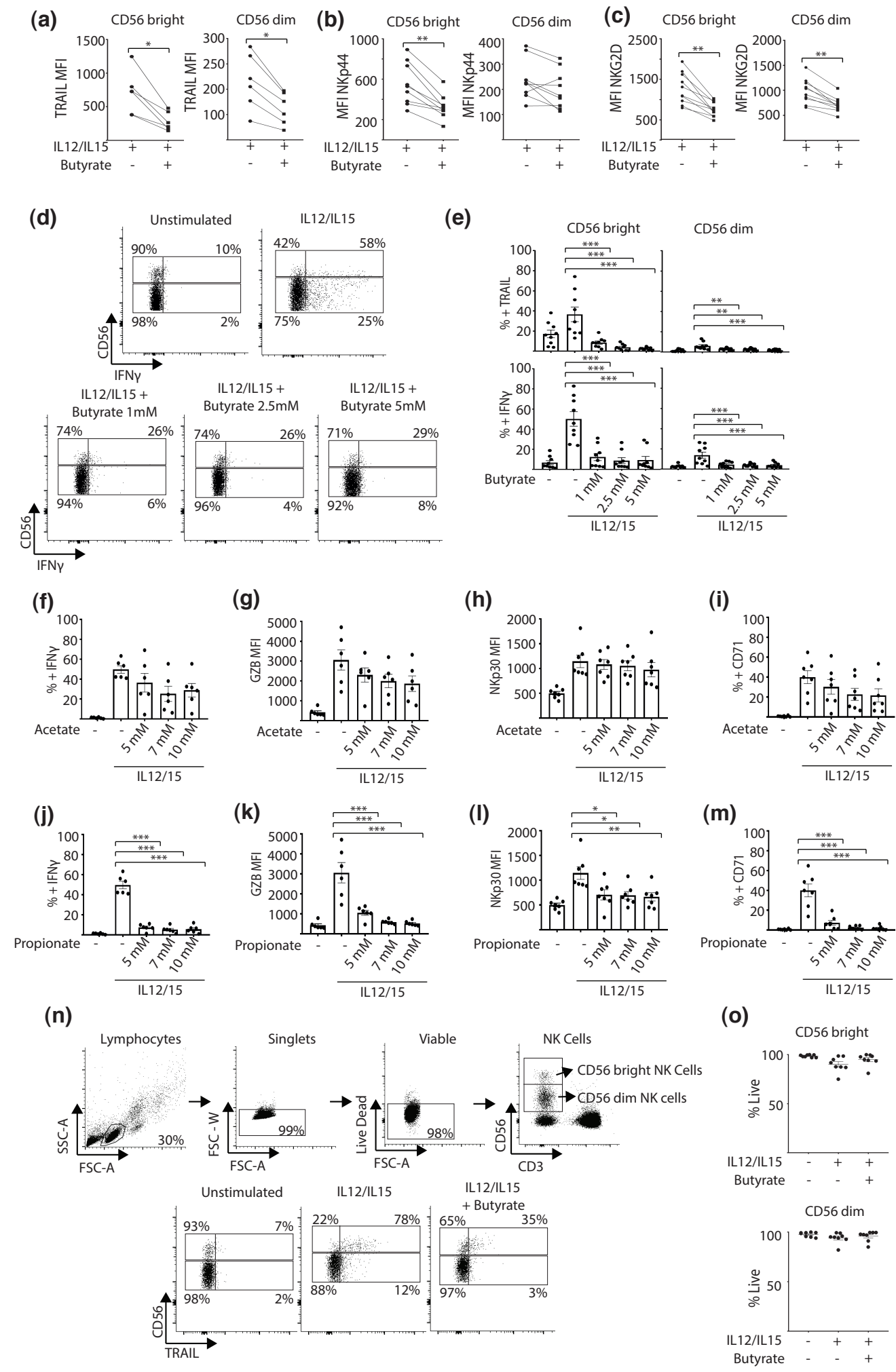

Supplement: Supplementary file 2 — Supplementary Figure 1. [file 41598_2023_29731_MOESM2_ESM.pdf]

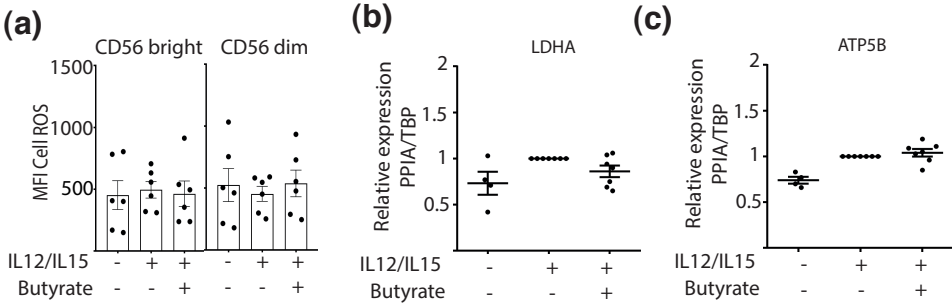

Supplement: Supplementary file 3 — Supplementary Figure 2. [file 41598_2023_29731_MOESM3_ESM.pdf]
